# Socio-demographic associations of HIV among women attending antenatal care in selected rural primary care facilities in South Africa’s Eastern Cape province

**DOI:** 10.1186/s12879-020-05744-7

**Published:** 2021-01-13

**Authors:** Sikhumbuzo A. Mabunda, Khuthala Sigovana, Wezile Chitha, Teke Apalata, Sibusiso Nomatshila

**Affiliations:** 1 The George Institute for Global Health and Research, University of New South Wales, Level 5 – 1 King Street, Sydney, Newtown NSW 2042 Australia; 2 Department of Public Health, Walter Sisulu University, Mthatha, South Africa; 3 Health Systems Enablement & Innovation Unit, University of the Witwatersrand, Johannesburg, South Africa; 4 Department of Laboratory Medicine, Walter Sisulu University, Mthatha, South Africa

**Keywords:** HIV, Antenatal, Prevention of mother-to-child transmission or PMTCT, South Africa

## Abstract

**Background:**

To effectively reduce vertical HIV transmission requires a reduction of HIV prevalence and incidence among pregnant women and a full understanding of its epidemiology. The study aimed to determine the prevalence of HIV among women attending antenatal care and further determine spousal support during antenatal care attendance in rural areas in Eastern Cape province, South Africa.

**Methods:**

A Cross-sectional study of women attending antenatal care in four Primary Care facilities was conducted using an interviewer-administered questionnaire which collected information on socio-demographic characteristics and medical history. Binomial logistic regression analyses were used to determine factors associated with HIV and to estimate the prevalence ratio (PR). The 95% confidence interval (95%CI) is used for precision of estimates; *p*≤0.05 for statistical significance.

**Results:**

A total of 343 participants were included in the final analysis. The antenatal HIV prevalence was 38.2% (95%CI: 33.2–43.9). For 75% of the women, the HIV diagnosis was made 141 days before the date of the interview (median=77 days, interquartile range=42–141 days). Participants between the age of 30 to 39 years were 50% more likely to be HIV positive compared to those who were between the age of 20 to 29, these differences were statistically significant (PR=1.5; *p*-value=0.001). Furthermore, self-employed women were 30% less likely to be HIV positive when compared to unemployed participants, this was also statistically significant (PR=0.7; *p*-value< 0.0001).

**Conclusion:**

Despite a 100% antenatal HIV testing rate, the antenatal HIV prevalence remains high in this population, coupled with no spousal attendance in antenatal care. It is important to move beyond awareness about the HIV status to actionable strategies of reducing the HIV incident cases. It is therefore important to remain vigilant and monitor mother-to-child transmission that could be associated with this increased prevalence.

## Background

Accounting for almost 25.5% of the total disease burden, South Africa’s HIV disease burden is four times higher than that of high-income countries [1–3]. In 2018, this translated to an estimated HIV population prevalence of 13.3% (~ 7.7 million people) [4]. The corresponding prevalence in women of reproductive age (15–49) was 20.4% [4]. More than a decade since the implementation of the Prevention of Mother-to-Child Transmission (PMTCT) programme of HIV, more than 90% of pregnant women are said to know their HIV status, the percentage of women living with HIV accessing antiretroviral treatment is estimated to have increased from 65% in 2010 to 87% in 2018 and there has been an 84% reduction in HIV infected newborns [4–9].

However, despite these strides and advances in HIV care including universal test and treat [10, 11], there is still persistence of incident maternal and newborn HIV infections [4–9]. Vertical transmission of HIV from mother to child can take place during pregnancy, delivery and lactation [12]. The World Health Organization (WHO) European regions especially the Eastern and Central sub-regions reported an increase of HIV infection in infants and children during the perinatal period from 347 to 494 per 1000 live births between the years 2004 and 2011 [13, 14]. It is not clear what the rates are currently.

In 2015, the estimated national HIV antenatal prevalence for South Africa was 30.8%, which is the highest estimate recorded in the preceding 5-years [15]. Even though high in the same period, the Eastern Cape province exhibited a decline in its antenatal HIV prevalence where it showed signs of stabilisation since 2005, having increased by only 0.7% from 1990 to 2015 [15]. Over the 5-year period (2011–2015), the point prevalence estimate reached a peak in 2013 and 2014 at 31.4% (95%CI: 29.4–33.5%); and declined by 1.2% in 2015 to 30.2% [15].

In women, the HIV risk has always been known to be decreasing with increasing age [1–3, 14–17]. According to Stats SA [17], approximately 20% of South African women in their reproductive years (15–49 years) are HIV positive; however, HIV prevalence among those aged 15–24 has declined over time from 7.3% in 2002 to 4.6% in 2017 [17]. A previous 10-year trends analysis (2003 to 2013) in South Africa’s KwaZulu-Natal Province among pregnant women previously showed declining HIV prevalences among teenagers and increased significantly among women 30 years and older [18].

Such high prevalences are of concern as they hinder efforts for an HIV free generation. These high HIV prevalences have been previously associated with economic dependence on the partner, age disparities of partner(s), sex under the influence of alcohol, inconsistent condom use, and having multiple sexual partners [19, 20]. Effective HIV prevention among women in antenatal care therefore needs incorporation of the biopsychosocial approach to HIV care [19].

To effectively reduce the HIV incidence in newborns, requires an increased HIV testing uptake among pregnant women, a reduction of HIV prevalence amongst pregnant women, a suppressed viral load and a full understanding of the HIV epidemiology among pregnant women. Every good policy needs continued monitoring, comparisons from different implementation sites and sharing of lessons that will lead to refinement [21]. With all the policy changes in HIV care, assumptions are that the epidemiology of HIV would have also been affected, e.g. reduction in incidence, improved testing, reduced stigma, etc. This has however, been found to not have been the case as demonstrated by the increasing HIV prevalences among pregnant women and the inability to achieve an HIV free generation as yet [5–8, 18, 19, 22].

The research aimed to determine the HIV antenatal prevalence, socio-demographic associations of HIV and the extent of the male partner attendance of antenatal care. Study findings will provide valuable information to health providers and will assist in prioritising, planning and strengthening of the PMTCT programme.

## Methods

### Study design

This quantitative cross-sectional study included all pregnant women who had at-least one antenatal visit, who used antenatal care services at the OR Tambo and Chris Hani Districts in the Eastern Cape Province, South Africa between March and November 2016. The period of enrolment was chosen to ensure that all women had been offered at least one HIV test (including those who refused) and had results available in their antenatal care records. Furthermore, this ensured that their partners had a chance for at-least one antenatal visit before interviews. The HIV status of initially negative participants was re-assesed at 34–37 weeks of pregnancy to ascertain a change in status from the initial interview. HIV positive participants were further recruited into a cohort study for the HIV status of their newborns to be established at 6 & 10 weeks. However, this article is only limited to findings on the HIV status of women attending antenatal care and its socio-demographic determinants.

### Study setting

The Eastern Cape Province is one of nine of South Africa’s Provinces. This is the Province with the second biggest surface area, the third most populous and the most rural with eight health districts [23]. Two of these (OR Tambo and Chris Hani) were purposefully selected for this study due to being the districts with the second and third highest HIV prevalences in the Eastern Cape Province [24]. Two facilities from each of these two districts were purposefully selected due to the high headcounts [24]. The province was chosen for this study due to its rurality, size and high HIV prevalence.

### Study design, population and sampling

Study Participants were recruited from four community health centres. Participants under the age of 18 were included after providing written consent from both themselves and their parents or guardians. All other pregnant participants issued a voluntary informed, written consent to participate.

All pregnant women attending antenatal care who were present in the facility on the day of the visit were recruited into the study if they met the inclusion criteria until the sample size was reached. Facilities were weighted at 48, 34, 9 and 9% based on their headcounts.

Using this equation ($n=\frac{p\left(100-p\right){z}^2}{d^2}$), a one-sided 95% confidence interval and a 5% significance level (z= 1.96), an estimated antenatal HIV prevalence of 29.5% (p) [16] and a desired precision (d) of 5%, a minimum sample size of 320 participants was calculated. An addition of 10% (*n*=32) to give allowance for non-responses, yielded a desired sample size of 352 participants. Nine participants were excluded due to missing date of birth (9) with either spousal information (7) and/or condom use information (8).

### Measurements

A validated interviewer administered questionnaire that was adapted from three instruments that have previously been used to measure HIV prevalence, PMTCT effectiveness and HIV stigma in developing countries [25–27]. It obtained information on socio-demographic characteristics, perception of health status and the medical history including HIV status (main outcome). The HIV status, the gestational age and antenatal care history were confirmed from the antenatal care card. Social desirability bias and language bias were mitigated through the use of a validated instrument, semi-private interviewing space, training of researchers on professional conduct during interviews (e.g. phrasing of questions, avoidance of gestures, etc.) and the translation of the questionnaire into the local language (isiXhosa) respectively. The instrument was piloted among 12 pregnant women in the four study sites. This pilot allowed for the refinement of the instrument before data collection.

### Statistical analysis

Stata version 14.1 (STATA Corp, College Station, Texas, USA) was used to analyse data. Considering the low levels of missing data, missing data are analysed using complete case analysis. Numerical variables were explored for normality using the Shapiro Wilk test [28]. Numerical data were not normally distributed and thus reported on using the median and Interquartile Range (IQR). The Wilcoxon Sum rank test (Mann-Whitney U test) was used to test for the equality of two medians, e.g. age in years by HIV status. Categorical variables are presented using, frequency tables, percentages and graphs. Two categorical variables were compared using the Chi-squared test if the expected frequencies were ≥ 5. The Fisher’s exact test was used for this purpose if the expected frequencies were < 5 as was the case in the comparison between the HIV and marital status.

Binomial logistics regression was used to determine the associations of an HIV positive status and to estimate the Prevalence Ratio (PR). The univariable models and the multivariable model selected through the purposeful selection of variables are presented [29]. This process ensured that the model selected is the best fit and adjusted for confounding and relevant covariates. The clustered sandwhich estimator and the intraclass correlation tests were used to assess for clustering by primary care facility. The 95% Confidence Interval (95%CI) was used to estimate the precision of estimates. The level of significance was set at 5% (*p*-value ≤ 0.05) for statistical significance.

The Walter Sisulu University Human Ethics and Biosafety Committee granted ethical clearance with ethics approval number (052/2016). The Eastern Cape Provincial Health Research Committee granted research access approval (EC_2016RP27_272).

## Results

Socio-demographic characteristics, HIV status and duration of HIV diagnosis of participants are presented in Table 1. A total of 343/352 participants (97.4%; 95%CI: 95.2–98.8) were included in the final analysis, of whom 38.2% (*n*=131) were HIV infected. Four of the nine (4/9 or 44.4%) of the excluded participants were HIV positive. There was no significant difference in the HIV status of participants in the four health facilities. HIV positive participants (median age=30) were significantly older than HIV negative participants (median=25; *p*-value< 0.0001). The youngest participants were 15 years old, with 35 teenagers (10.2%) and the oldest were 43 years. High school learners and tertiary students comprised 33 (9.6%) and 32 (9.3%) of participants respectively. All participants knew their HIV status and the main reasons for having an HIV test included the fact that it was mandatory in antenatal care (44.0%); for health reasons or tests as a routine (47.2%); for sake of unborn baby (5.3%) and 3.5% reported to have tested due to being medically unwell.

**Table 1:** Socio-demographic characteristics (*N*=343)

Demographics and Medical characteristics	HIV infected		HIV uninfected		***p***-value
	***N***=131		***N***=212		
HIV status; n (%)	131	(38.2)	212	(61.8)	< 0.0001
Age, years; median (IQR^a^)	30	(10)	25	(9)	< 0.0001
Duration of HIV diagnosis (*N*=127), days; median (IQR)	77	(99)	–		–
^c^Gestational age at booking, weeks (*N*=339); median (IQR)	15.1	(11) ^d^	16	(8) ^e^	0.419
Age, years; n (%)					
15–19	8	(22.9)	27	(77.1)	< 0.0001
20–29	53	(28.8)	131	(71.2)	
30–39	60	(53.6)	52	(46.4)	
40–43	10	(83.3)	2	(16.7)	
Facility; n (%)					
Ngangelizwe CHC	61	(36.3)	107	(63.7)	0.672
Mhlakulo CHC	44	(37.9)	72	(62.1)	
Ngcobo CHC	14	(48.3)	15	(51.7)	
All Saints Gateway clinic	12	(40.0)	18	(60.0)	
First Pregnancy; n (%)					
No	100	(45.7)	119	(54.3)	< 0.0001
Yes	31	(25.0)	93	(75.0)	
Marital Status; n (%)					
Married	35	(44.9)	43	(55.1)	0.037^b^
Never Married	84	(34.3)	161	(65.7)	
Cohabiting	9	(60.0)	6	(40.0)	
Divorced	1	(33.3)	2	(66.7)	
Widowed	2	(100.0)	0	(0)	
Current Education; n (%)					
High School Learner	8	(24.2)	25	(75.8)	< 0.0001
Tertiary Student	3	(9.4)	29	(90.6)	
Not currently studying	120	(43.2)	158	(56.8)	
Employment; n (%)					
Employed	33	(47.8)	36	(52.2)	0.662
Unemployed	83	(41.7)	116	(58.3)	
Self-employed	4	(40.0)	6	(60.0)	
Spouse in high school; n (%)					
Yes	2	(6.1)	31	(93.9)	< 0.0001
No	129	(41.6)	181	(58.4)	
Spousal occupation; n (%)					
Locally Employed	64	(41.8)	89	(58.2)	0.210
Employed in another town	42	(38.2)	68	(61.8)	
Unemployed	22	(53.7)	19	(46.3)	
Self-employed	1	(16.7)	5	(83.3)	

^a^IQR = Interquartile Range = 75th percentile – 25th percentile

^b^Fisher’s Exact test was used

^c^This is the estimated gestational age at first antenatal care reading based on clinical records

^d^*n* = 209; ^e^*n*=130

The median gestational age at antenatal care booking for the 339 (98.8%) respondents who had complete information was 15.6 weeks (IQR: 11.3–20.3) and there was no significant difference between HIV positive and HIV negative individuals. Whilst one patient had been on HAART for 13-years, at-least 80.3% (*n*=102) of the participants had their HIV status diagnosed in the index pregnancy, with a median duration of diagnosis and initiation on HAART of 77 days (IQR: 42–141).

Primigravidas accounted for 124 (36.1%) of all participants and there were statistical differences between HIV positive and negative participants (*p*-value< 0.0001). All 2 widowed participants, 9/6 (60.0%) of cohabiting participants, 35/78 (44.9%) married participants and 84/245 (34.3%) of participants who were never married had an HIV positive diagnosis and this was statistically significant (*p*-value = 0.037). Whilst 120/278 (43.2%) of those who were currently studying were HIV positive, 8/33 (24.2%) of those in high school and 3/32 of those in tertiary were HIV positive, this was statistically significant (*p*-value< 0.0001). Unemployed participants accounted for 199 (58.0%) of all participants, of which 83/199 (41.7%) were HIV positive. Spouses were distributed between those employed locally (44.6%), employed in another town (32.1%), unemployed (12.0%), high school learners (9.6%) and self-employed (1.8%). Only 2/33 (6.1%) of those whose spouses were in high school were HIV positive. Of the participants whose spouses were unemployed, 53.7% (*n* = 22/41) were HIV positive.

**Fig. 1:**
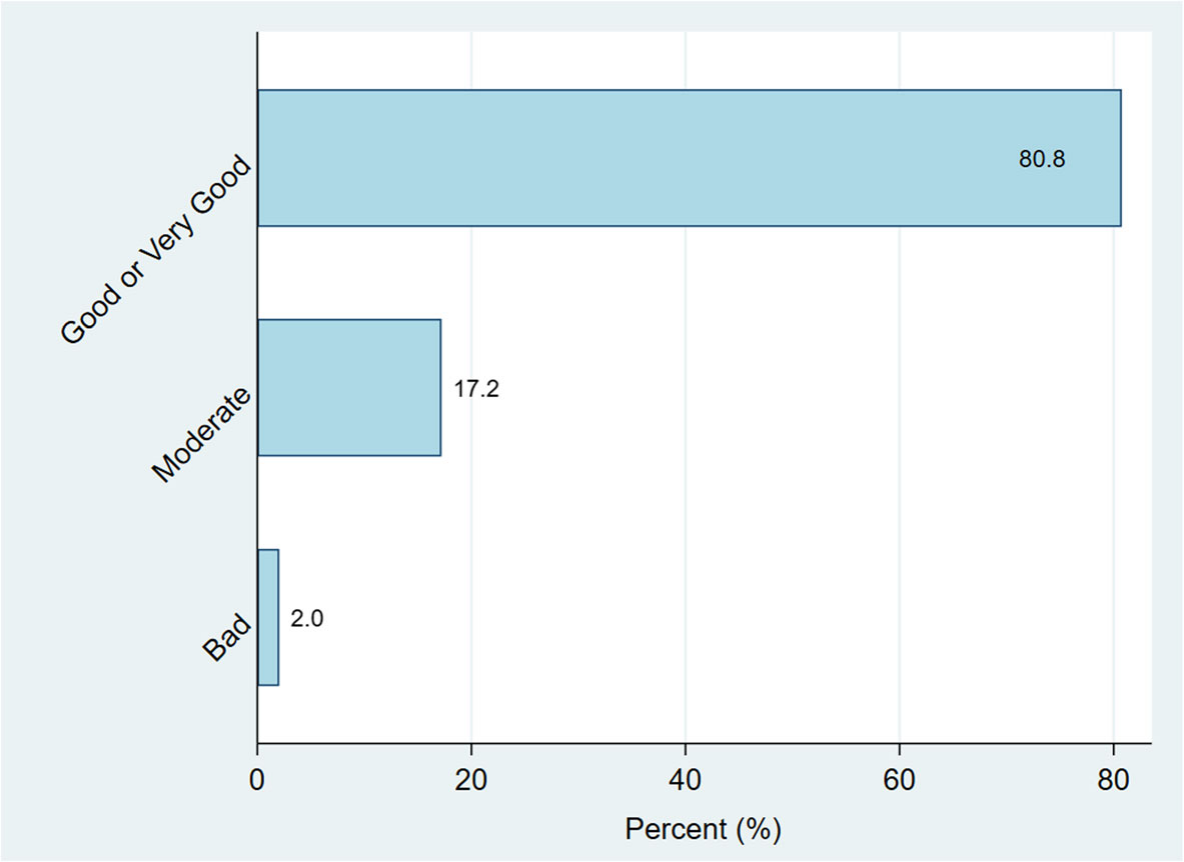
Perceived Health Status

**Fig. 2:**
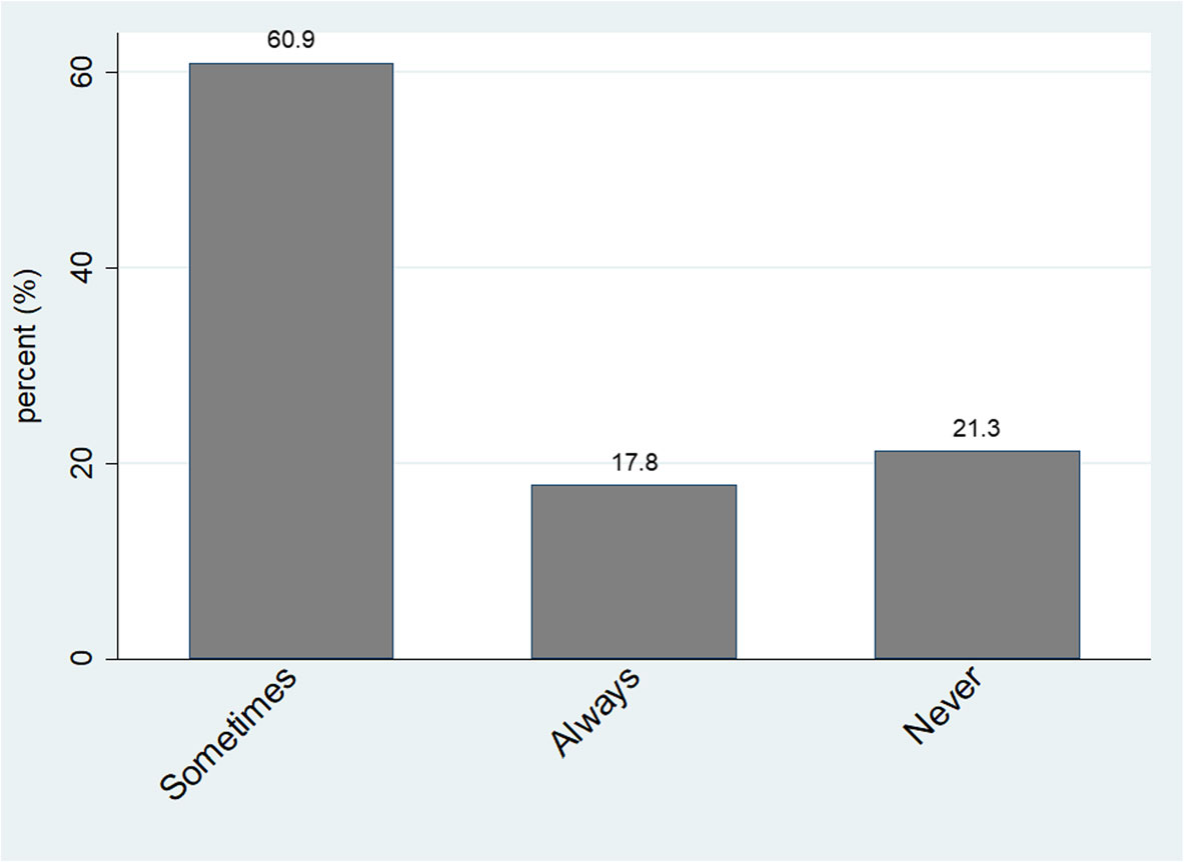
Reported Condom Use

Paricipants reported their health status to be very good or good (80.8% or *n*=277), moderate (17.2% or *n*=59), or bad (2.0% or *n*=7) respectively (Fig. 1). Whilst 209 (60.9%) participants reported to condomise sometimes, 73 (21.3%) reported to never and 61 (17.9%) reported to always condomise (Fig. 2). Only 72/73 respondents (98.6%) stated reasons for never using a condom. One respondent (1.4%) never used a condom because the index pregnancy was her sexual debut. Others cited reasons such as being married (3/72 or 4.2%); trusting partner (13/72 or 18.1%); partner refusal (33/72 or 45.8%); sexual preference (21/72 or 29.2%) and one respondent (1.4%) associated condom use with swelling or rash in the genital area.

**Fig. 3:**
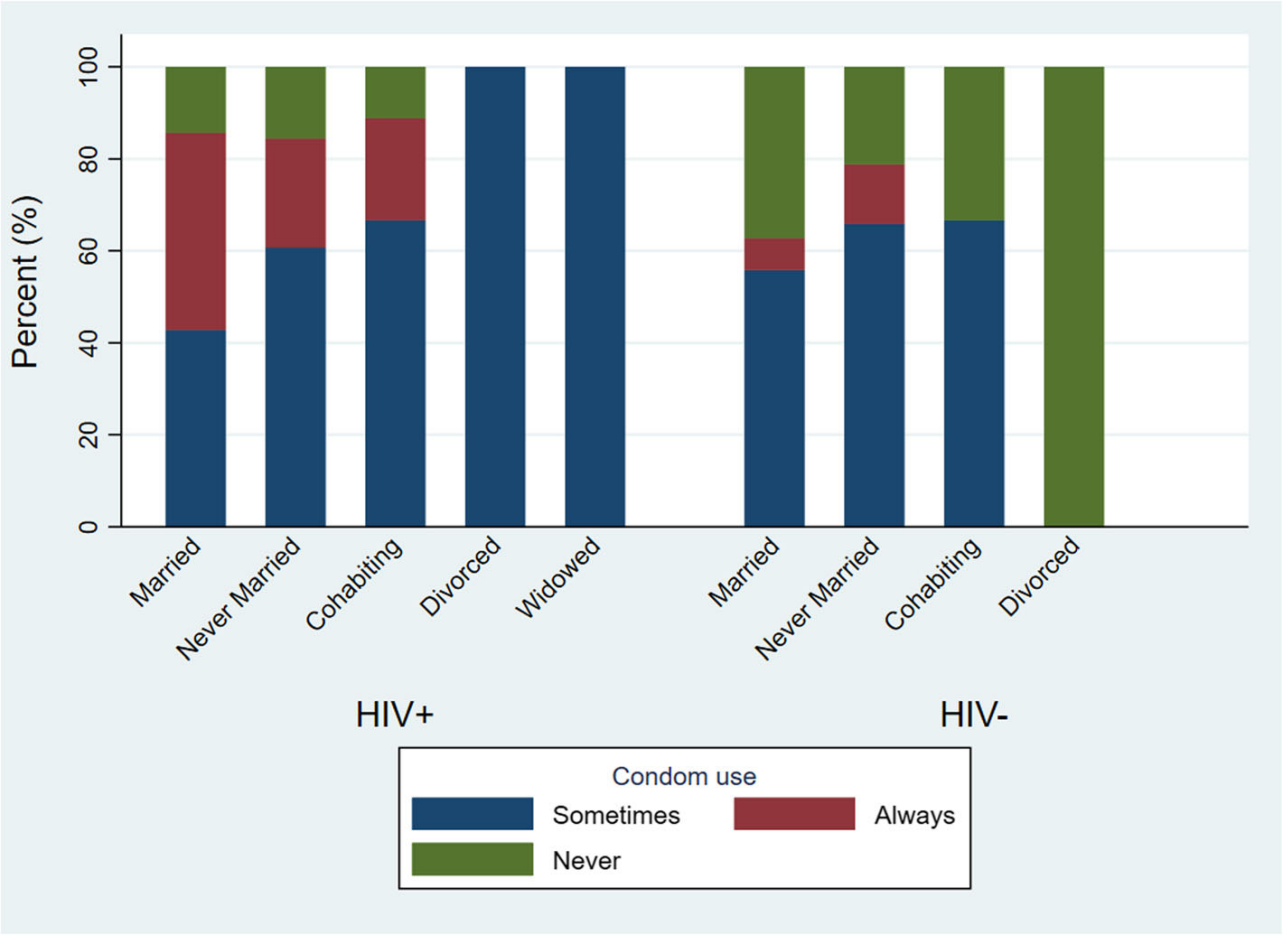
Frequency of Condom use by HIV and Marital status

Of the married HIV positive participants, 15/35 (42.9%) reported to either use condoms all the time or sometimes (Fig. 3). Only three (3/43 or 7.0%) HIV negative participants who were married reported to always use condoms, the majority (55.8% or 24/43) reported to condomise sometimes.

Only 73 (21.3%) participants’ partners were reported to accompany them to antenatal care. Most participants’ partners (92.4%) were, however, reported to show an interest on the pregnancy. Partners did not attend antenatal care due to no longer being together (*n*=1); they stayed far apart (37.4%); other commitments (41.9%); had never discussed it (8.9%); partner refused to come (5.9%) and 5.6% believed that males were not welcome in antenatal care.

In univariable analysis, the antenatal HIV prevalence was significantly higher in participants between the ages of 30–39 (PR = 1.5; *p*-value< 0.0001) and 40–43 (PR = 4.3; *p*-value = 0.025) compared to those in the 20 to 29 year age group (Table 2). In addition, multigravida participants were associated with 40% higher antenatal HIV prevalences respectively when compared to primigravidas and this was statistically significant (PR = 1.4; *p*-value < 0.0001). Other factors associated with a significantly higher antenatal prevalence included participants who were not full-time students (PR = 1.6; *p*-value< 0.0001) and the spouse being unemployed (PR = 1.8; *p*-value = 0.018).

**Table 2:** Antenatal HIV Associated Factors

Characteristics	n	Univariable analysis			Multivariable analysis		
		PR (95% Confidence interval)		***p***-value	PR (95% Confidence interval)		***p***-value
Age, years							
20–29	53/184	ref		1	ref		1
15–19	8/35	0.9	(0.8–1.1)	0.437	1.0	(0.7–1.4)	0.988
30–39	60/112	1.5	(1.2–1.9)	< 0.0001	1.5	(1.2–1.9)	0.001
40–43	10/12	4.3	(1.2–15.2)	0.025	3.6	(1.0–12.6)	0.048
First pregnancy							
Yes	31/124	ref		1	ref		1
No	100/219	1.4	(1.2–1.6)	< 0.0001	1.1	(0.9–1.4)	0.329
Employment Status							
Unemployed	83/199	ref		1			
Employed	33/69	0.9	(0.7–1.2)	0.393	1.0	(0.8–1.2)	0.848
Self-employed	4/10	1.0	(0.6–1.6)	0.913	0.7	(0.6–0.8)	< 0.0001
Current Education							
Tertiary student	3/32	ref		1	–		–
High school learner	8/33	1.2	(1.0–1.5)	0.115	–		–
Not currently studying	120/278	1.6	(1.4–1.9)	< 0.0001	–		–
Perceived Health status							
Good/Very Good	77/218	ref		1	–		–
Moderate	18/59	0.9	(0.7–1.1)	0.187	–		–
Bad	5/7	2.4	(0.7–7.9)	0.141	–		–
Spouse in high school							
Yes	2/33	ref		1	–		–
No	129/310	1.6	(1.4–1.8)	< 0.0001	–		–
Spousal Employment							
Self-employed	1/6	ref		1	–		–
Locally employed	64/153	1.1	(0.9–1.3)	0.549	–		–
Employed in another town	42/110	1.3	(0.9–2.0)	0.130	–		–
Unemployed	22/41	1.8	(1.1–2.9)	0.018	–		–

*PR* Prevalence Ratio, *ref* reference

Multivariable analysis had three variables in the best fitting binomial logistic regression model (Table 2). Gravidity was not a statistically significant association of antenatal HIV prevalence (*p*-value=0.329). Those in the 30 to 39 year age group were 50% more likely to be HIV positive and this was statistically significant (PR = 1.5; *p*-value = 0.001). Likewise, those in the 40 to 43 year age group were 3.6 times as likely to be HIV positive when compared to those in their 20s and this difference was borderline statistically significant (PR = 3.6; *p*-value = 0.048). However, self-employed participants were 30% less likely to be HIV positive when compared to unemployed participants and this was statistically significant (*p*-value< 0.0001).

## Discussion

This study will hopefully add evidence to the already existing body of knowledge on South Africa’s HIV epidemiology, especially among pregnant women in rural communities. It is a study which highlights a high HIV antenatal prevalence, high antenatal HIV testing rates, lack of expectation of spousal attendance in antenatal care and challenges experienced with condom compliance. In univariable analysis, multigravidity, being older than 30 years and having an unemployed spouse was associated with higher HIV prevalences, confirming findings that have previously been established in literature [5, 9, 18–20, 22, 30]. The study does not only provide an update of the antenatal HIV prevalence but also seeks to use epidemiological data to inform health promotion practices in a rural South African environment. Lessons from this high HIV burden country will hopefully also be applicable to other LMIC and their planners. The major difference between this study and the South African antenatal surveys is that this study includes all pregnant women and not exclusively primigravidas [15, 16].

Of the 10.2% of teenagers interviewed in the study, 94.3% were high school students and 22.9% (*n*=8) were HIV positive. This teenage pregnancy rate is lower than that described by Mchunu et al. [31] in a similar South African population wherein 19.2% of women reported to have fallen pregnant during their teenage years [31]. The HIV prevalence among teenagers is comparable to a prevalence of 17.2 to 22.5% reported in a 10 year cohort in a neighbouring South African province of KwaZulu-Natal [18]. Regardless of the percentage of teenagers who were pregnant, it cannot be ideal for school children who are themselves dependent on adults to be pregnant as this often has an impact on their long-term progress [31, 32].

This is a poverty-stricken community with more than half of the women interviewed being unemployed (57.7%), never married (71.4%) and multiparous (63.9%). Almost 45% of the women’s partners were employed locally, suggesting that there were economic opportunities locally that favour males. These compare to other antenatal care survey results such as that in another South African province (Limpopo), where 808 pregnant women were recruited, 51% from rural areas and 28% from peri-urban areas [33]. In that study both rural and peri-urban pregnant women had a high rate of being unemployed and being unmarried [33]. The fact that self-employed women were less likely to be HIV positive in the multivariable analysis (*p*-value< 0.0001) is consistent with findings suggesting that economic independence could improve the capacity to negotiate safer sexual practices [19]. The results have to however be treated with caution given that self-employed participants only accounted for 10 (2.9%) of participants in the study.

Encouraging is the fact that 50% of women had their first antenatal care visit at 16 weeks. This is good as it allows adequate time for identification of congenital abnormalities, maternal or foetal risks and the suppression of the viral load if HIV positive, thus reducing the probability of Mother-to-Child-Transmission [13, 14, 22, 34–36]. This compares to antenatal care survey results of a Cameroonian study [37], of 293 pregnant participants, where 34% had started antenatal care in the first trimester [37]. The explanations most commonly offered for a late antenatal presentation were financial difficulties and living a long way from the health facility [37].

The data shows that antenatal HIV prevalence is increasing and higher than that presented in previous studies for same area [15]. The crude antenatal HIV prevalence of 38.2% is higher than the 31.9% (95%CI: 27.4–36.8) and the 33.3% (95%CI: 30.4–36.4) antenatal prevalences previously reported for Chris Hani and OR Tambo Districts respectively [15]. The differences could be attributed to the inclusion of multigravida women in this study [15, 16]. The high prevalence could be a result of an increasing incidence most probably related to poor condom compliance and the concurrent reduction of HIV related mortality due to an improved antiretroviral programme [17, 18, 30]. The HIV status of participants was not dependent on the facility from which they were recruited, and this was not statistically significant (*p*-value = 0.672). Findings from this study contrast the 2015 South African National Antenatal Sentinel HIV and Syphilis survey results which reported a declining HIV prevalence for the Eastern Cape Province [15].

Pregnancy in early adolescence has been found to be associated with an increased incidence of HIV infection among South African women [38]. The higher risk is associated with sexual risk behaviour such as multiple partners and a greater age difference with partners [38]. This study, however, found a different phenomenon: the prevalence is higher amongst older women which suggests changes in the epidemiological characteristics possibly since the HIV infected women were infected many years previously but only knew their HIV positive status in the index pregnancy or it could well be a mark of an increasing incidence among older women. The aim of PMTCT programs is to improve the wellbeing of expectant mothers and to reduce the incidence of HIV among newborns [39, 40]. Future retrospective cohort studies should seek to quantify the HIV MTCT trends among newborns in the same study population, especially since multigravidas were associated with a higher prevalence than primigravidas.

It is of little surprise that unemployed women had a significantly higher risk of being HIV positive than self-employed women. HIV is a disease of poverty [41], which further explains the increasing HIV prevalence in this community where more than 50% of the participants were unemployed [41]. Poverty may drive some women into risky sexual behaviours such as transactional sex and an inability to negotiate safer sexual practices with their partner [41].

Most women did not have an expectation for their partners to accompany them during their antenatal care visits. Spousal support during antenatal care can help improve acceptance and utilisation of preventive strategies in general and to an increased uptake of interventions to prevent vertical and sexual transmission of HIV [42]. Partner/couple counselling in the antenatal setting may have further benefits to individual VCT [42]. In a Kenyan study, male antenatal care attendance was found to be associated with improved infant HIV-free survival [43]. Promotion of HIV testing in men and engagement in antenatal care services may improve outcomes in infants [43].

The non-involvement of partners in antenatal care services could discourage women in their ability to disclose their HIV-positive status due to fear of rejection, stigma and discrimination. It could also serve as a barrier to women beginning treatment and adhering to it and may disrupt HIV prevention services which could in turn result in poor HIV outcomes.

Even though attempts were made to reduce limitations the study encountered a few. Firstly, findings from this study are not representative of the Eastern Cape Province as participants were recruited from only four health facilities. Findings from this study do, however, give a reasonable idea of the epidemiology of HIV in a rural environment amongst women attending antenatal care. Secondly, the strength of the association between women older than 40 years and those between 20 to 29 years is weak due to the borderline *p*-value and the wide confidence interval.

Thirdly, the limited privacy during the interviews that occurred as a result of infrastructure challenges could have resulted in a social desirability bias, especially in questions pertaining to sexual behaviour and the use of condoms. Where this bias was noted results are reported truthfully. It is however unlikely that these limitations could have distorted the findings on the epidemiology of HIV in this population especially since medical information was triangulated from clinical records.

## Conclusion

A successful PMTCT program is bolstered by an early antenatal care attendance and high HIV testing rates. This was evident in this study with a median antenatal booking attendance of 15.6 weeks of gestation and an HIV testing rate of 100%. The study has shown an antenatal HIV prevalence of 38.2% and found to be higher among older and multigravida women.

This population showed a very low uptake of spousal attendance in antenatal care as it was not an expectation for spouses to attend. Antenatal care nurses and policymakers should therefore make extra effort for spouses to be ecourage to attend antenatal care.

## Data Availability

The datasets used and/or analysed during the current study are available on request from the corresponding author on request.

## Abbreviations

ARVs: Antiretrovirals
CHC: Community Health Centres
HAART: Highly Active Antiretroviral Therapy
LMIC: Low- and middle-income countries
MTCT: Mother-to-Child Transmission
NCDs: Non-communicable diseases
PMTCT: Prevention of Mother-to-Child Transmission
PR: Prevalence Ratio
TB: Tuberculosis
UNAIDS: The Joint United Nations Programme on HIV and AIDS
VCT: Voluntary Counselling and Testing
WHO: World Health Organization
